# Cutting edge concepts: Does bilirubin enhance exercise performance?

**DOI:** 10.3389/fspor.2022.1040687

**Published:** 2023-01-11

**Authors:** Kyle D. Flack, Libor Vítek, Christopher S. Fry, David E. Stec, Terry D. Hinds

**Affiliations:** ^1^ Department of Dietetics and Human Nutrition, University of Kentucky, Lexington, KY, United States; ^2^ 4th Department of Internal Medicine and Institute of Medical Biochemistry and Laboratory Diagnostics, 1st Faculty of Medicine, Charles University and General University Hospital in Prague, Prague, Czechia; ^3^ Department of Athletic Training and Clinical Nutrition, University of Kentucky College of Medicine, Lexington, KY, United States; ^4^ Center for Muscle Biology, University of Kentucky College of Medicine, Lexington, KY, United States; ^5^ Department of Physiology & Biophysics, Cardiorenal, and Metabolic Diseases Research Center, University of Mississippi Medical Center, Jackson, MS, United States; ^6^ Department of Pharmacology and Nutritional Sciences, University of Kentucky College of Medicine, Lexington, KY, United States; ^7^ Barnstable Brown Diabetes Center, University of Kentucky College of Medicine, Lexington, KY, United States; ^8^ Markey Cancer Center, University of Kentucky, Lexington, KY, United States

**Keywords:** exercise performance, heme oxygenase, biliverdin reductase, bilirubin, reactive oxygen species, oxidative stress, HO-1, BLVRA

## Abstract

Exercise performance is dependent on many factors, such as muscular strength and endurance, cardiovascular capacity, liver health, and metabolic flexibility. Recent studies show that plasma levels of bilirubin, which has classically been viewed as a liver dysfunction biomarker, are elevated by exercise training and that elite athletes may have significantly higher levels. Other studies have shown higher plasma bilirubin levels in athletes and active individuals compared to general, sedentary populations. The reason for these adaptions is unclear, but it could be related to bilirubin's antioxidant properties in response to a large number of reactive oxygen species (ROS) that originates from mitochondria during exercise. However, the mechanisms of these are unknown. Current research has re-defined bilirubin as a metabolic hormone that interacts with nuclear receptors to drive gene transcription, which reduces body weight. Bilirubin has been shown to reduce adiposity and improve the cardiovascular system, which might be related to the adaption of bilirubin increasing during exercise. No studies have directly tested if elevating bilirubin levels can influence athletic performance. However, based on the mechanisms proposed in the present review, this seems plausible and an area to consider for future studies. Here, we discuss the importance of bilirubin and exercise and how the combination might improve metabolic health outcomes and possibly athletic performance.

## Introduction

Exercise training can promote the physiological health of every organ system in the body, carrying a myriad of benefits, including improving blood glucose control, cardiovascular capacity, arterial compliance, skeletal muscle function, and energy metabolism (1–4). In fact, 35 chronic diseases or conditions have been independently linked to physical inactivity (5). Most health outcomes of regular exercise, such as improving aspects of the metabolic syndrome, depend on skeletal muscle adaptations (6). However, recent data has pointed to exercise-induced benefits in liver metabolism and function playing a vital role (7–9). Exercise increases hepatic glycogen mobilization when exercise bouts are sustained beyond short bursts of high-intensity activity that rely on intramuscular stores of glucose and fat (9, 10). As hepatic glycogen is reduced with extended exercise, the liver is also responsible for the uptake of gluconeogenic precursors such as lactate, pyruvate, ketones, and glycerol (10–13). This is accomplished, in part, by exercise-induced reductions in lipogenic processes and a simultaneous increase in the lipid oxidation (14, 15), a potential mechanism for how exercise can prevent liver diseases such as non-alcoholic fatty liver disease (NAFLD) (16, 17). Interestingly, a classical liver disease biomarker, bilirubin (11), has been shown to increase with exercise (18). Studies also show that increasing bilirubin levels decreases liver fat content and reduces oxidative stress in obese mice, improving adiposity and blood glucose (19–22). Other work has shown that aerobic exercise protects the liver and cardiometabolic health and adipose tissue remodeling under metabolic stress (23). These adaptations might be linked to glucose and fatty acid metabolism during exercise, which points to well-controlled crosstalk between the liver and skeletal muscle, exchanging substrates and maintaining metabolic homeostasis. Thus, exercise-induced adaptations centered on improving substrate utilization, also termed metabolic flexibility, are not solely dependent on the skeletal muscle metabolism (9, 10).

Exercise can also play an important role in weight control by aiding in attaining an energy deficit and the metabolic adaptations in the glucose and fatty-acid metabolism (24, 25). Although other aspects of metabolic syndrome can be improved with exercise alone (without weight loss), these benefits are substantially greater when significant weight loss occurs (26). While we later discuss that plasma bilirubin levels are elevated with exercise, another facet is that it also increases during weight loss (27). With the continually prevalent obesity epidemic, exercising for weight loss will continue to be a prevailing theme in research trials. It will be interesting to see whether bilirubin will be a measurable component of future works, especially since it has many protective properties that reduce oxidative stress.

An additional adaptation to exercise that may influence substrate utilization is the upregulation of antioxidant defense systems (18, 28, 29); this is partially due to increased ROS, and reactive nitrogen species (RNS) observed with exercise (30, 31). Such free radical production during exercise can have key regulatory roles in mediating various signaling processes. However, when increases in free radicals are not met with increases in antioxidant defense, pathophysiological states such as inflammatory, cardiovascular, and neurodegenerative diseases may manifest (32). Recent research has focused on oxidative stress and exercise mechanisms, with many exploring the utility of additional antioxidant supplementation when engaging in consistent exercise (18, 33, 34). New findings have revealed that the antioxidant bilirubin may be significantly elevated in athletes (18, 35). Other recent works have shown that bilirubin has a hormonal function that reduces body weight and may be related to exercise capacity (19, 36–44). These findings point to bilirubin as an underlying mediator of exercise-induced alterations in substrate oxidation, weight loss, antioxidant status, and a surrogate to the aforementioned health outcomes (37, 38, 42, 44). Herein, we will delve into the recent literature investigating the link between bilirubin, exercise, and physiological health.

## Bilirubin and exercise

Traditionally viewed as a marker for liver damage, bilirubin is becoming recognized as an important endocrine hormone and a potent antioxidant that activates nuclear receptors to control gene transcription that promotes many aspects of physiological health (cardiovascular health, blood glucose control, oxidative stress, and improves liver function) (37, 39, 43, 45). The medical community has defined “normal” total plasma bilirubin levels as 1.7–20 µmol/L, while the Child-Pugh index indicates a value of >51 µmol/L is indicative of decompensated liver cirrhosis. Large variations in plasma bilirubin are exhibited among the general population due to age, sex, ethnicity, and other biological factors. Thus, it is difficult to define a particular range for other non-clinical conditions such as long-term exercise, acute exercise, obesity, and lean individuals (46). The concept of hypobilirubinemia has been recently proposed at levels of plasma/serum bilirubin <10 µmol/L [discussed further in (37)].

**Figure 1:**
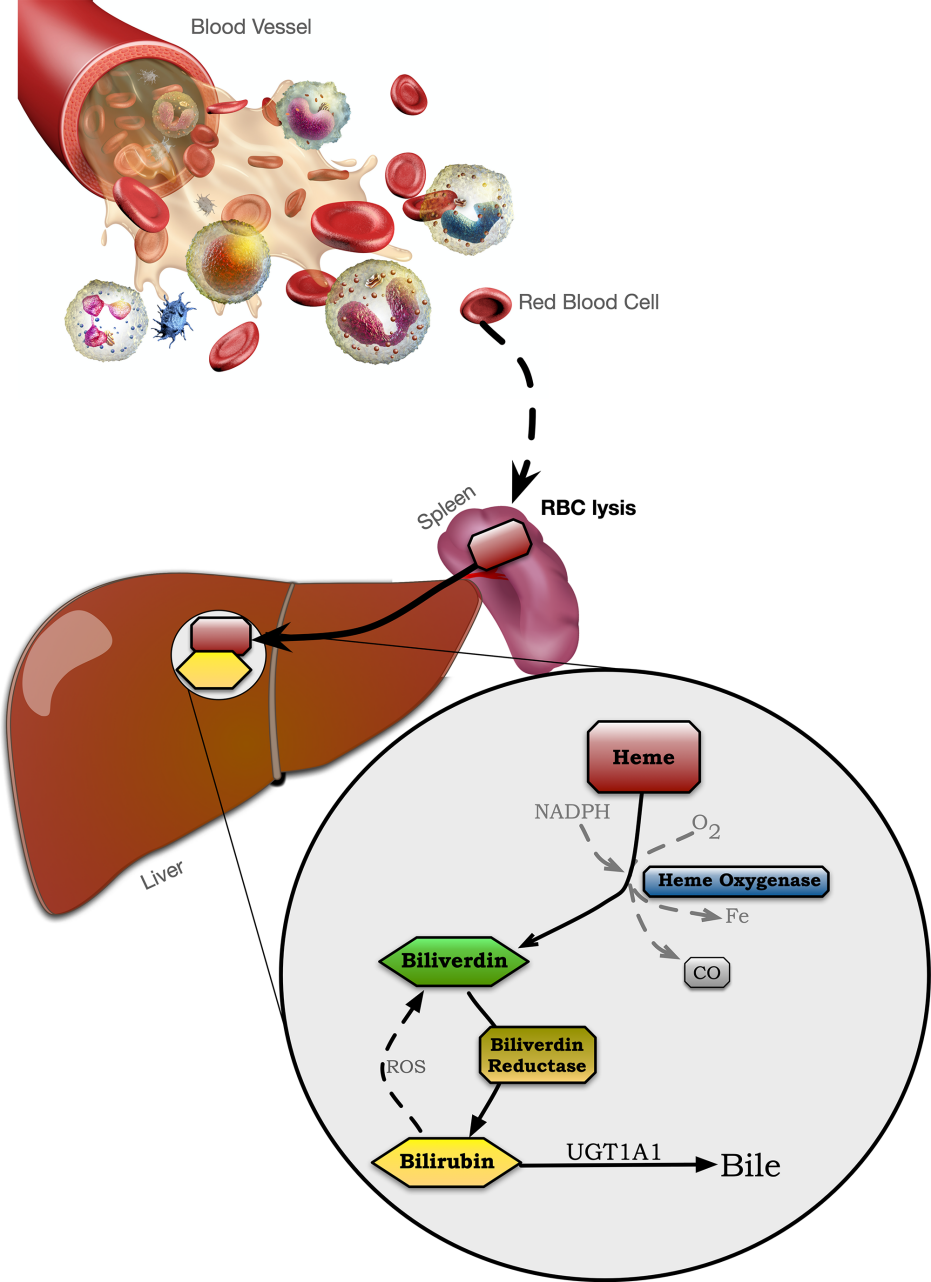
The heme oxygenase-bilirubin pathway.

Bilirubin originates from hemoglobin released from myoglobin and other hemoproteins during the destruction of senescent red blood cells. When a blood cell dies and is lysed, which occurs mostly in the spleen, heme is released and converted to biliverdin by heme oxygenase (HO), which is further metabolized to bilirubin by biliverdin reductase A (BVRA) (Figure 1) (47). Blood bilirubin levels have previously been thought only to be derived from reticuloendothelial cells in the spleen (37). However, studies in mice lacking BVRA (21, 48–50) have shown that bilirubin generation also occurs in many other tissues. Lastly, bilirubin is conjugated by the UDP-glucuronosyltransferase enzyme, UGT1A1 (51), which then deposits the conjugated bilirubin in the bile (43). Thus, it is possible to regulate plasma bilirubin levels by regulating HO, BVRA, or UGT1A1. Recently published work showed that high-capacity running rats (HCR), compared to low-capacity running rats (LCR), had significantly higher plasma bilirubin, which was likely due to hepatic BVRA being raised and UGT1A1 lowered (52). These ultimately cause higher bilirubin production by BVRA and less bilirubin clearance *via* UGT1A1 conjugation.

Although research connecting bilirubin and exercise is in its infancy, a limited number of studies have demonstrated that bilirubin may be increased with both acute and regular (long-term) endurance exercise in animal models and humans (52–55). This was observed in the Dose-Response to Exercise in Women Trial (DREW Trial), where participants were placed in three groups of varying exercise volumes (4, 8, or 12 kcal.kg.week) for 12 weeks, demonstrating bilirubin only increased in the 12 kcal.kg.week group, equivalent to an average of 169 min per week (54). This dose-response relationship is supported by a separate trial where 12 weeks of exercise training that progressed to 120 min per week did not influence bilirubin levels (56). Thus, exercise meeting or slightly exceeding the recommended 150 min of moderate to vigorous physical activity per week appears necessary to observe physiological (beneficial) increases in the plasma bilirubin (57). This is also supported in less controlled trials, where bilirubin increases after 3 months of soccer or rugby training in competitive athletes (58, 59) and is elevated in competitive athletes compared to the general population (35, 60). Associations have also been drawn between usual exercise behavior, where aerobic and strength training participation was positively related to plasma bilirubin levels among women. In contrast, only aerobic training participation was positively correlated in the men (61). There is also evidence that an acute bout of exercise (often exhaustive) can upregulate plasma bilirubin. This was demonstrated in trained and untrained adults and adolescents after a running time trial test to exhaustion (62). A maximal exercise test also increased plasma bilirubin among football players (63) and was increased 4 days after an ultra-marathon among trained runners (64).

**Figure 2:**
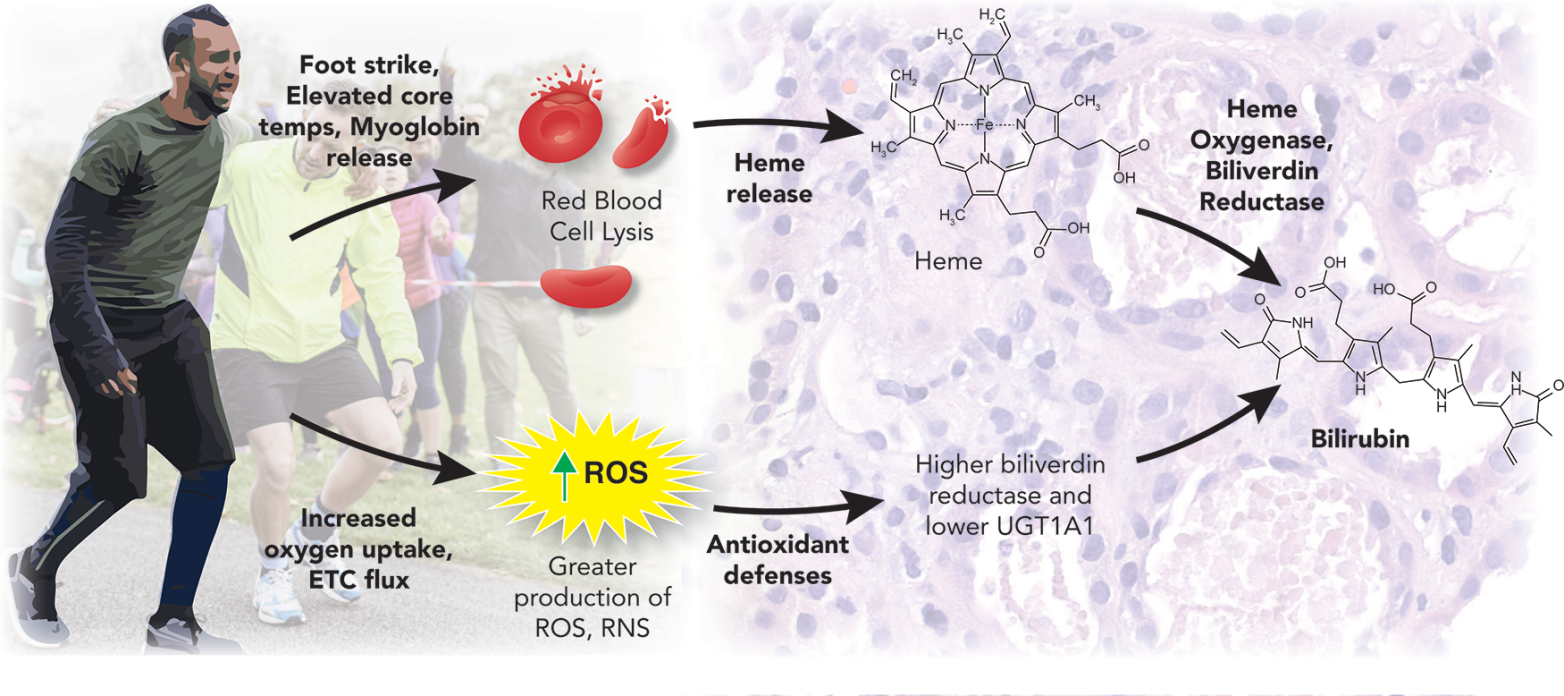
Exercise and bilirubin production. This figure was created by Matthew Hazzard at the University of Kentucky College of Medicine.

An important question yet to be fully elucidated is the mechanisms induced by exercise that cause the reciprocal increase in plasma bilirubin (Figure 2). One theory is that heme catabolism could result from exercise (especially aerobic exercise) induced damage such as repeated foot strikes, elevated core temps, and skeletal muscle breakdown (myoglobin release) (65, 66). In this scenario, red blood cells may become lysed and release heme (hemolysis). This released heme can be broken down to biliverdin by heme oxygenase-1 (HO-1) and further catabolized by BVRA to eventually form a stable, unconjugated bilirubin (43). This view is supported by several of the findings above, where only the highest dose of exercise, which had the greatest exposure to factors associated with exercise-induced hemolysis, observed increases in the plasma bilirubin (54). This logic could also be applied to trained athletes exposed to very high levels of factors that may induce hemolysis to promote the observed elevations in bilirubin levels (35, 58–60). However, Swift et al. did not detect changes in hemoglobin or hematocrit following exercise training (54). This has been supported by Witek et al.'s work on athletes, who concluded that the hematological parameters did not indicate the occurrence of increased hemolysis, with no significant relationship between the total bilirubin concentration and the number of red blood cells, hemoglobin, or iron levels in the blood of trained athletes (60). These results are similar to those of Andelkovic et al., where 3 months of soccer training did not increase serum iron (likely to reflect hemolysis) nor transferrin (likely to reflect erythropoiesis due to increased hemolysis) (58). Although this study also demonstrated increased serum ferritin after training and positive correlations between bilirubin and ferritin post-training (58). Since ferritin is known to sequester iron in the blood, increased ferritin levels may mask the elevations in iron resulting from exercise-induced hemolysis; an antioxidant adaptation of ferritin has been previously demonstrated (67, 68). However, this has not been consistent across studies, with many showing no changes or decreases in ferritin after long-term exercise training in athletes’ (69–71). Other arguments against exercise-induced hemolysis driving greater bilirubin levels seen in athletes or after a long-term intervention are the notion that markers of hemolysis are typically present only immediately after intense exercise (63, 66), which would support why plasma bilirubin can increase after a bout of acute exercise (62–64).

Another hypothesis is that exercise-induced increases in bilirubin are the result of a feedback mechanism to regulate the increased oxidative stress that accompanies physical training (35). As noted, bilirubin is a powerful antioxidant, and if following other antioxidant defense systems, it should increase with long-term exercise training to better control exercise-induced free radical damage (18, 28, 29). Indeed, the long-term exercise effect on bilirubin is associated with an increase in other antioxidant reserves as well, including total antioxidant status (35). Such increases in bilirubin would likely result from greater HO activity, which is increased with exercise training (72). Since HO is the rate-limiting enzyme necessary for converting heme to biliverdin (73), greater HO levels could force the observed increase in plasma bilirubin after long-term exercise or in physical activity individuals/athletes. Other mechanisms promoting an exercise-induced increase in plasma bilirubin could involve the enzyme that converts biliverdin to bilirubin (BVRA) (74) or the enzyme that is responsible for the removal of bilirubin from the blood into bile (UGT1A1). As noted, HCR mice demonstrated higher plasma bilirubin and increased BVRA expression while UGT1A1 was decreased compared to control animals (52). Interestingly, hepatic HO-1 was not different between the HCR mice and control, despite large differences in distance and time run to exhaustion. This indicates that exercise-induced increases in bilirubin can stem from changes in several different enzymes, including HO-1, BVRA, and UGT1A1 (Figure 1). It seems likely that long-term adaptations to exercise training promote antioxidant defenses, including bilirubin, while short-term adaptations include those related to exercise-induced damage and increased heme availability.

## Gilbert’s syndrome and exercise

Although it seems that regular physical training leads to an elevation in serum bilirubin concentrations, additional considerations need to be given to Gilbert's Syndrome (GS), a genetic polymorphism that reduces UGT1A1 expression, increasing plasma bilirubin levels to potentially influence athletic performance (53). This has been demonstrated in elite Czech athletes, where elite sportsmen and sportswomen had significantly greater serum bilirubin concentrations (8.5–16 µmol/L) compared to the general population (53). At the same time, the prevalence rate of phenotypic GS syndrome was also much higher in elite athletes, suggesting that a mild elevation of serum bilirubin might predispose to better sports performance. In other words, mildly hyperbilirubinemic elite athletes could have been selected based on this biochemical trait to reach the sport's elite. This provides further evidence that bilirubin may promote athletic performance, likely related to bilirubin's role as an endocrine hormone, inducing gene transcription that modulates metabolic functions. Increased systemic concentrations of bilirubin may represent a feedback mechanism to:

a) cope with the increased oxidative stress that accompanies the training process (30),
b) provide signaling stimuli to the muscle (75) and cardiovascular system (76), improve adaptation to physical training stress, and simultaneously,
c) provide substantial metabolic advantages regarding fatty acid oxidation associated with regular exercise (77).

These conclusions are based on recent observations. Regular exercise has been associated with increased antioxidant capacity, similar to a previous report documenting an exercise-induced increase in other body antioxidant reserves (62). In addition, bilirubin is an important signaling molecule (78, 79), fulfilling parameters of the endocrine substance (45). Therefore, these activities are highly likely to contribute to the beneficiary metabolic adaptations associated with regular training.

## Bilirubin and cardiovascular system as a benefit for exercise

**Figure 3:**
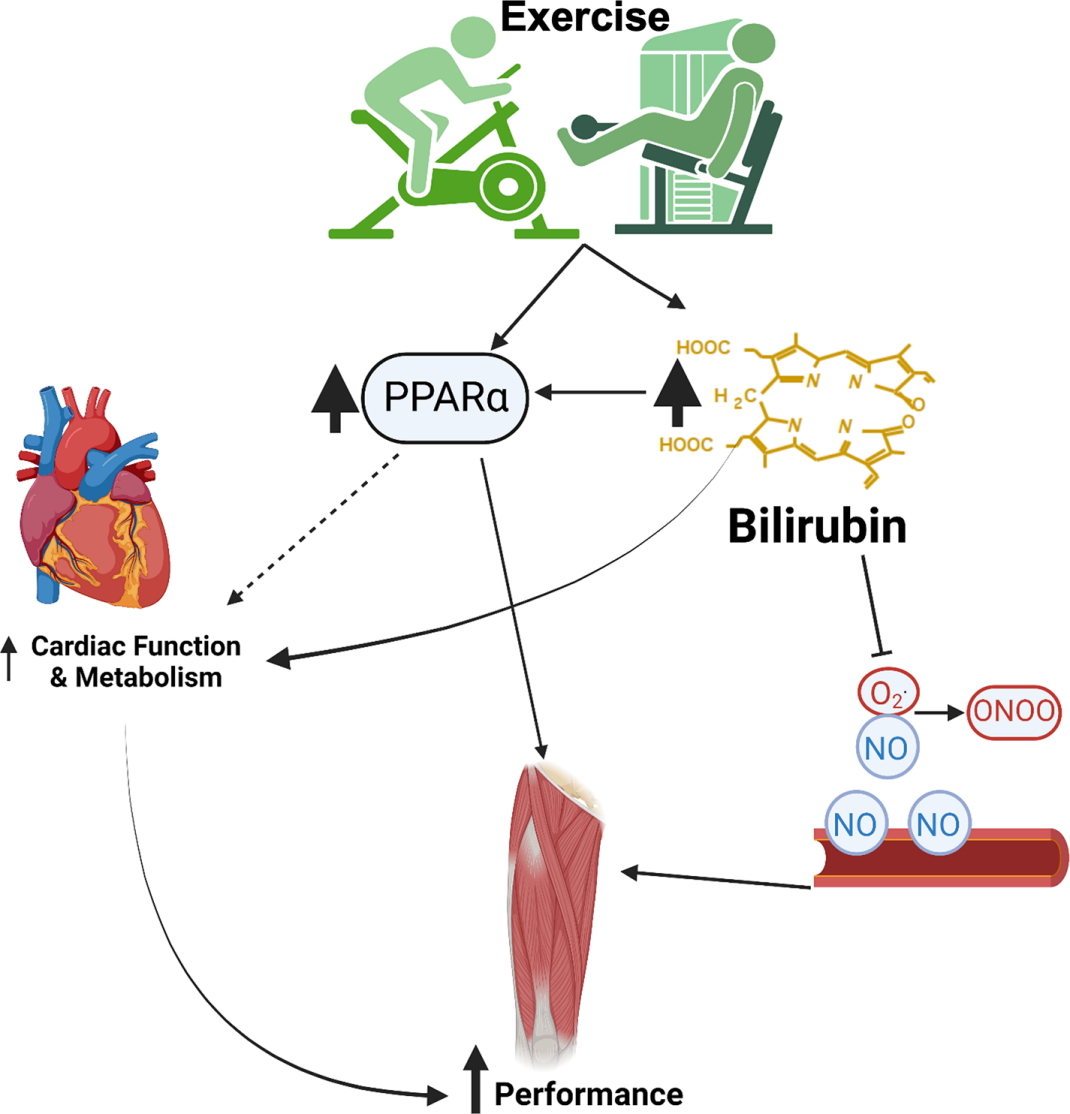
Cardiovascular benefits of exercise and bilirubin. This image was made using Biorender.com.

Increased plasma bilirubin levels can have several beneficial effects on the cardiovascular system in the context of exercise (Figure 3). First, bilirubin is a potent antioxidant compound that can scavenge ROS both directly and through the inhibition of the NAD(P)H oxidase (80, 81). One of the main targets of the ROS product superoxide anion (O_2_^•^) is nitric oxide (NO). Superoxide interacts with NO to form peroxynitrite radical, damaging DNA and nitrosylate tyrosine residues, which disrupts protein function. By limiting the production and actions of superoxide, bilirubin can increase the bioavailability of NO to preserve the blood flow (82–84). The preservation of blood flow through enhanced NO bioavailability may mediate the improvement in athletic performance observed with increased levels of plasma bilirubin (35). Bilirubin mimics the protective actions of HO-1 induction and restores attenuated eNOS expression after exposure to oxLDL and TNF-α (85). The hyperbilirubinemic Gunn rat is resistant to the pressor actions of angiotensin II, and bilirubin can attenuate the release of endothelin-1 (86, 87). These findings demonstrate that bilirubin has vasoprotective actions, which could be beneficial to maintaining blood flow during exercise.

Recent studies have indicated that bilirubin functions as a hormone to activate the nuclear receptor peroxisome proliferator-activated receptor alpha (PPARα). It has been proposed that low plasma bilirubin levels should be considered a pathological state (37, 44). PPARα activation in the liver is a contributory factor to the exercise-related improvements in the whole-body metabolism (88). In fact, induction of PPARα in the vasculature by exercise has recently been proposed as a therapy to fight COVID-19 infection (89). Gene polymorphisms in PPARα increase physical and aerobic performance and are associated with muscle fiber type composition in athletes’ (90, 91). Twice a day, close proximity exercise is associated with enhanced mitochondrial biogenesis, fat oxidation, and upregulation of skeletal muscle PPARα (92). Likewise, treatment with the PPARα agonist, fenofibrate, increases soleus muscle weight and enhances musculoskeletal training response during estrogen deficiency in ovariectomized (OVX) Sprague Dawley rats (93). Exercise training also decreases the age-related decline in cardiac PPARα levels in rats (94). PPARα knockout mice exhibited reduced lipolysis and anti-inflammatory responses in adipose tissue following exercise (95). The adipose-specific PPARα KO (96) and liver-specific PPARα KO (97) animals exhibited adiposity in the null tissues, which further indicates the importance of the bilirubin-PPARα circuit.

PPARα affects changes in metabolism central to exercise adaptation and muscle stem cell dynamics. Satellite cells, the *bonafide* muscle stem cell, support skeletal muscle exercise adaptation through activation and fusion into muscle fibers (98–100). Exercise-induced satellite cell activation is reliant on dynamic metabolic reprogramming culminating in robust activation of oxidative metabolism during the terminal differentiation (101). PPARα is a critical regulator of muscle lipid homeostasis to facilitate differentiation of human satellite cells *in vitro* to support subsequent fusion into muscle fibers to facilitate exercise-induced adaptation (102, 103). Furthermore, skeletal muscle is a mosaic of different fiber “types” uniquely defined by their metabolic requirement. The targeting by PPARα of genes involved in cellular fatty acid import and binding help define a unique cellular identity for PPARα in oxidative type I fibers versus the predominantly glycolytic type II muscle fibers (104). Greater demand for mitochondrial biogenesis and oxidative metabolism that occur in response to chronic exercise supports a fiber type-specific role for PPARα-mediated transcription. Variance in human type I fiber distribution is closely associated with PPARα expression, offering further support for PPARα in the distinct metabolic requirements of oxidative, slow twitch type I fibers (105). Further studies in PPARα deficient animals are needed in order to fully elucidate the role of PPARα activation in response to increases in bilirubin production in exercise.

Bilirubin is also cardio-protective, and increased bilirubin levels during exercise may benefit the heart. For example, studies in hyperbilirubinemic Gunn rats demonstrate that bilirubin protects the heart from reperfusion injury and beneficially influences aortic ejection velocities and pressures, improving cardiac performance during exercise (106, 107). Recent studies have demonstrated that bilirubin can increase the production of hepatic ketone beta-hydroxybutyrate (BOHB) (19), which likely occurred *via* PPARα mechanisms. A diet supplemented with BOHB precursors improved exercise performance in rats (108). Ketones may play an important role in the metabolic adaptation of the heart to exercise, especially in type II diabetic patients who are unable to effectively utilize glucose as a cardiac energy source. While the protective actions of bilirubin on the heart have largely been explained through its potent antioxidant activity, the effects of bilirubin on cardiac metabolism remain to be thoroughly studied. It is possible that bilirubin plays an important role in the metabolic adaptation of the heart to exercise both directly and indirectly through its action on PPARα and hepatic production of BOHB.

There is mounting evidence pointing to bilirubin as an important hormonal molecule and antioxidant, a departure from the traditional view that the role of bilirubin was limited to a marker for liver dysfunction. Bilirubin's role in mediating metabolic adaptations and protecting from oxidative stress is now evident, most notably in the context of cardiovascular disease and obesity. The present review has further explored the role exercise training appears to have on bilirubin levels, outlining two primary metabolic pathways activated by exercise that promote slight elevations in plasma bilirubin. The first of these pathways are related to heme catabolism, where exercise-induced damage such as muscle strain causing myoglobin release, elevated core temperature, and repetitive foot strike causes red blood cell lysis and heme release. Using heme as a precursor, through actions of the HO and BVRA enzymes, bilirubin synthesis is increased. This pathway seems to be impacted primarily by acute exercise, as reductions in hemolysis can be a long-term training adaptation observed among athletes (69–71). An additional pathway that can increase exercise-induced elevations in plasma bilirubin is related to an upregulation of antioxidant defense mechanisms. Just as other antioxidant enzymes are increased in response to elevations in ROS and RNS that accompany exercise (62), including total antioxidant status (35), BVRA can be increased while the enzyme UGT1A1 is decreased, thus promoting the synthesis and increased plasma levels of bilirubin (52). The combination of both pathways explains how both long-term and acute exercise can promote bilirubin levels and why athletes have consistently demonstrated greater plasma bilirubin levels compared to the general population.

## Conclusion

Although no studies have directly tested if increasing plasma bilirubin levels promote improved exercise performance, this hypothesis seems probable with the evidence presented and an area for future research exploration. Preliminary evidence that supports this hypothesis is related to studies on GS and elite athlete performance. These individuals have a specific genetic polymorphism that causes elevated plasma bilirubin, where a far greater prevalence of GS is observed in elite athletes. This suggests that individuals with greater bilirubin levels might be predisposed to greater athletic performance. This could be related to bilirubin's role as a hormonal signaling molecule, where bilirubin interacts with PPARα to stimulate gene transcription related to fatty acid oxidative and mitochondrial capacities, important mediators in muscle function and exercise performance (102, 103). Improving antioxidant defenses through elevations in bilirubin is also desirable for athletic performance, likely related to enhanced bioavailability of NO and increased blood flow (35, 82–84). Controlled trials in humans testing the potential utility of bilirubin playing an ergogenic role in exercise performance are lacking and, thus, an additional avenue for future investigation. The optimal level of plasma bilirubin has also not been defined for health or athletic performance, another important question that research may address. Altogether, future work to determine whether increasing plasma bilirubin levels are useful for enhancing athletic performance is needed before research can focus on ergogenic aids to increase plasma bilirubin. In the least, bilirubin is an important molecule and new hormone that improves metabolic function and could be an essential metabolite of exercise performance and weight loss.
